# Kinetic Modeling Reveals a Common Death Niche for Newly Formed and Mature B Cells

**DOI:** 10.1371/journal.pone.0009497

**Published:** 2010-03-02

**Authors:** Gitit Shahaf, Michael P. Cancro, Ramit Mehr

**Affiliations:** 1 The Mina and Everard Goodman Faculty of Life Sciences, Bar-Ilan University, Ramat-Gan, Israel; 2 Pathology and Laboratory Medicine, University of Pennsylvania School of Medicine, Philadelphia, Pennsylvania, United States of America; University of Miami, United States of America

## Abstract

**Background:**

B lymphocytes are subject to elimination following strong BCR ligation in the absence of appropriate second signals, and this mechanism mediates substantial cell losses during late differentiation steps in the bone marrow and periphery. Mature B cells may also be eliminated through this mechanism as well as through normal turnover, but the population containing mature cells destined for elimination has not been identified. Herein, we asked whether the transitional 3 (T3) subset, which contains most newly formed cells undergoing anergic death, could also include mature B cells destined for elimination.

**Methodology/Principal Findings:**

To interrogate this hypothesis and its implications, we applied mathematical models to previously generated in vivo labeling data. Our analyses reveal that the death rate of T3 B cells is far higher than the death rates of all other splenic B cell subpopulations. Further, the model, in which the T3 pool includes both newly formed and mature primary B cells destined for apoptotic death, shows that this cell loss may account for nearly all mature B cell turnover.

**Conclusions/Significance:**

This finding has implications for the mechanism of normal mature B cell turnover.

## Introduction

Following immunoglobulin (Ig) gene rearrangement and the expression of a functional B cell receptor (BCR) (reviewed in [1]–[4]) in the bone marrow (BM), immature (IMM) B cells exit to the periphery as transitional (TR) B cells, where they complete maturation and then enter the follicular (FO) or marginal zone (MZ) pools [5]–[8]. While the elimination of autoreactive B cells can occur at any differentiative stage after functional BCR expression [9]–[16], most tolerogenic death is believed to occur at the IMM and TR stages, inasmuch as these are the first expressing a functional BCR, and cells within these subsets seem predisposed to BCR-induced death [11]–[12], [15], [17]–[18]. Consistent with this view, in vivo labeling studies have revealed that under steady state conditions, only about half a million of the roughly fifteen million IMM BM B cells produced daily survive to join the mature peripheral pools [19]–[20]. About 90% of these losses occur via deletion at the IMM BM stage. The remaining losses occur through anergic cell death, whereby cells engaged in low-avidity interactions survive to enter the TR stages but die before completing maturation [21]–[22].

The notion that anergic cells reside briefly in the TR compartment before dying, as well as the belief that mature cells are also subject to tolerogenic elimination if their BCR is engaged without costimulation, prompts several questions. First, whether particular TR phenotypes correspond to cells undergoing apoptotic death versus those that will complete maturation is unclear. Second, if particular phenotypes correspond to dying cells, the proportional contributions of newly formed versus mature cells to these pools require definition. Since mature B cells are non-dividing, the relatively rapid turnover of TR pools suggests that most losses in these subsets reflect the death of recent marrow émigrés. Nonetheless, recent studies in transgenic systems have suggested that FO cells dying from lack of costimulation re-acquire the T3 phenotype [23], suggesting that this is characteristic of cells undergoing anergic death, and implying that at least some of the T3 pool is derived from mature B cells.

We have previously shown that mathematical modeling of population kinetics established from in vivo bromodeoxyuridine (BrdU) labeling studies is a powerful tool with which to assess alternative models of B cell differentiation and fate [24]–[27]. Our previous study of the population transitional B cells [26] has compared all possible models which include the linear differentiation pathway: bone marrow immature → T1 → T2 → T3 → Follicular mature B cells. When set out to perform that study, the exact progenitor–successor relationships of these transitional subsets, as well as whether a proliferative step is requisite for follicular B cell maturation, were controversial. Moreover, whether late B cell differentiation might involve branched or asynchronous maturation pathways, thus allowing some cells to ‘skip’ one or more of these stages, was also unknown. Hence, in that study, we have used mathematical modeling to interrogate these possibilities. Using mathematical models that numerically simulate each model of splenic B cell population dynamics and fit them to the experimental data, we have determined which models best fit the in vivo labeling data. The results indicate that follicular differentiation does not involve a proliferating splenic intermediate. Those same results further suggested that some developing cells move directly from the immature marrow pool to more advanced semi-mature peripheral subsets without passing through the least mature subset in the spleen.

**Figure 1:**
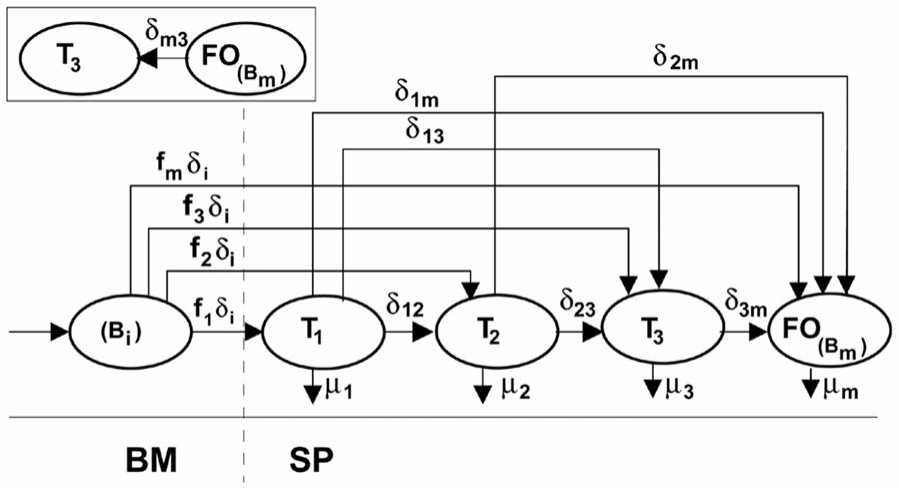
The alternative models of developing B cell populations in the spleen. The main figure shows the one found as the best model in our previous study [28]. The new hypothesis differs from our previous model only in the direction of flow between T3 and mature B cells, as shown in the inset. Cell subsets and parameters represented in our model are shown (see “Methods” for details).

In the present study, we ask whether T3 B cell compartment contains most peripheral B cell slated for elimination, and whether a model based on this hypothesis (The inset in Figure 1) can explain the quantitative relationship between T3 and mature B cells. We addressed these questions by fitting our mathematical models of B cell population kinetics in the BM and in the spleen [25]–[27], implementing this new hypothesis of T3 behavior, to the BrdU labeling data of Allman et al [28]. The results suggest that the T3 B cell subset is a major staging point for B cells undergoing apoptotic death, since this model can account for nearly all TR B cell losses. Further, our analyses show that including input from mature pools in the model yields a high fit for these data, with ∼40% of T3 throughput derived from the mature B cell population. Interestingly, this proportion could account for nearly all mature B cell turnover under the normal steady state, suggesting a common route and mechanism of loss from most primary B cell subsets.

## Methods

### Data for Model Fitting

In order to understand the behavior of the transitional B cell subpopulations that will become mature naive B cells in the spleen, we used published experimental data on these subpopulations in mice [28]. The data include measurements on four subpopulations that are the three transitional B cell subsets and the mature B cell subset in the spleen. Detailed methodological descriptions are available in [19], [28]. Briefly, mice were treated with i.p. injections of 0.5 mg bromodeoxyuridine (BrdU) (Sigma) twice daily. Splenocytes were analyzed at successive intervals by immunofluorescent staining for surface markers and incorporated BrdU. For each mouse, the percentage of BrdU-labeled cells in each subset was measured using flow cytometry and multiplied by the total cell number in the subset to give the total number of labeled cells. The values were plotted as a function of time.

### Mathematical Models

Our model starts with three bone marrow populations: pro-B [B220+CD43+IgM−], pre-B [B220+CD43−IgM−] and immature B cells [B220+HSA+IgM^hi^IgD^lo^], with cell numbers in these subsets represented by the variables Bo, Be and Bi, respectively. However, previous experimental observations distinguish between small, non-cycling cells and large, cycling cells in both the pro-B and pre-B compartments, where the transition from pro-B to pre-B occurs while the cells are cycling. Hence, we break the pro-B and pre-B subsets into two subsets each: Bor for small resting pro-B cells (Hardy's fractions A through C) and Boc for large cycling pro-B cells (part of Hardy's fraction C); similarly, Bec for large cycling pre-B cells (the remainder of fraction C) and Ber for small resting pre-B cells (fraction D). Immature B (Bi) cells migrate from the bone marrow to the periphery as transitional B cell. The four subpopulations in the periphery are the three transitional B cell subsets and the mature B cell subset in the spleen as defined by Allman et al, [28], Table 1.

**Table 1:** Post BCR-expression developmental subsets.

Anatomic Site	Status	Subset	Cycling?	Surface Phenotype
Bone marrow*	Immature	E	no	IgM^hi^IgD^lo^CD23^+/−^B220^+^AA4.1^+^
Periphery	Transitional [28]	T1	no	IgM^hi^CD23^−^B220^+^AA4.1^+^
		T2	no	IgM^hi^CD23^+^B220^+^AA4.1^+^
		T3	no	IgM^lo^CD23^+^B220^+^AA4.1^+^
	Transitional [33]	T1	no	IgM^hi^CD23-B220^+^IgD^−^CD21^−^HSA^hi^
		T2	yes	IgM^hi^CD23^+^B220^+^CD21^hi^IgD^lo^HSA^hi^
	Mature	FO/(B2)	no	IgM^lo^CD23^+^B220^hi^AA4.1^−^
		Marginal Zone(MZ)		CD9^+^IgM^hi^IgD^lo^CD23^−^CD21^+^
		B1		IgM^hi^CD43^+^IgD^lo/−^CD23^lo/−^

* This is the current accepted phenotype for immature B cells. Because we used historic data for these analyses, we felt it appropriate to indicate in the text the criteria used in the actual labeling studies, that is, B220+HSA+IgM^hi^IgD^lo^. It should be noted that this is unlikely to influence our conclusions, since the pro B subsets are not an immediate progenitor pool to any subsets that might enter the T3 pool, regardless of which model is used.

The numbers of cells in the T_1_ and T_2_ combined subsets, T_3_ and mature B cells were represented in our mathematical models by the variables *T_1/2_, T_3_* and *B_m_*. Bone-marrow cell populations were described, as in our previous study [25], by the following equations.
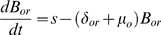
(1)

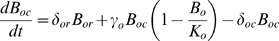
(2)

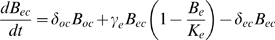
(3)

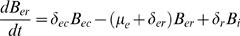
(4)

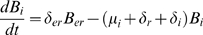
(5)


In these equations, the input of stem cells into pro-B compartment is denoted by *s* (for ‘source’), the parameters *δ* denote differentiation rates and the parameters *γ* denote proliferation rates. Proliferation of developing B cells is known to be limited by the finite space and resources (e.g. contact with the stroma, growth factors, nutrients) in the bone marrow [29]–[30]. Hence K_o_ and K_e_ denote the carrying capacities of the pro- and pre-B compartments, respectively, i.e. the population sizes for which the corresponding population growth rates become zero. Cell death is assumed in our model to occur only in the non-proliferating cell subsets, because proliferation, gene rearrangement and selection occur in distinct stages and cell death usually occurs only as a result of failure in the latter two processes. The corresponding population mortality rates are denoted by *μ*_o_, *μ*_e_, *μ*_i_ for B_or_, B_er_, B_i_ respectively. More information about this part of the model is found in [25].

Immature B (*B*_i_) cells migrate from the BM to the periphery with a constant rate of *δ_i_*. Out of the *δ_i_B_i_* cells that exit the BM daily, *f_1_* represents the fraction of these cells that differentiate to the *T_1/2_* combined subset. There may also be a fraction, *f_3_*, of cells that differentiate to *T_3_*, and a fraction, *f_m_*, of cells that differentiate to *B_m_*. *T_1/2_* cells differentiate to *T_3_* or to mature B cells (*B_m_*). We denote by *δ_23_* the differentiation rate of *T_1/2_* to *T*_3_, and by *δ_2m_* the differentiation rate of *T_1/2_* to *B_m_*. Based on previous studies we assumed that none of the transitional subpopulations are cycling [28], [26]. The exit/death rates from each compartment are denoted by *μ_1/2_*, *μ_3_*, and *μ_m_* for *T_1/2_*, *T_3_* and *B_m_*, respectively.

Finally, in the new model, no differentiation from *T_3_* into *B_m_* is allowed. Instead, a differentiation from *B_m_* into *T_3_*, denoted by *δ_m3_*, represents the new hypothesis that, when a mature naive B cell does not get a second activation signal from T cell, the cell then differentiates to the transitional 3 subset and dies.

The above-described hypothesis is described by the following equations.
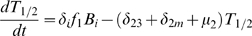
(6)

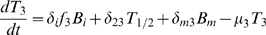
(7)

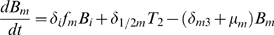
(8)


The numerical simulations of the mathematical models were performed in a program written in the C programming language, which runs on the entire parameter space in small intervals, searching for the best-fit parameter set for each model.

### Simulations

The mathematical models were simulated and fitted to data using a C language program. The program receives as input the experimental data, and the ranges of parameter values within which the model should be run. The program divides each range to very small intervals, thus providing a thorough coverage of the parameter space. This creates a set of 1.5×10^6^ parameter combinations to be checked by the program. For each parameter value set, the program integrates the model equations as follows. The initial conditions are zero cells in all populations; labeling starts after the populations have reached a steady state. After integration, the program first checks whether the total cell number and the fractions of cells in each population are within the experimentally measured ranges. Runs in which this is not the case are discarded. For all other runs, the program records the value of the fit of the model to the data (defined below), and outputs the parameter set(s) that have yielded the best fit. This process was performed for each of the models, and the fit values were compared using the AIC method (see below).

### Choosing the Best Model Parameters

In choosing alternative models and parameter values for the simulations of our model, we adhered to the following guidelines.

- The parameters should be in the experimentally observed orders of magnitude, if published information is available. While these estimates (where available) are usually not given in units of population rates, so that interpretation of most of these data depends on the model used, these estimates were useful in suggesting the appropriate value ranges for some of the parameters. For example, cell proliferation rates can not be higher than the equivalent of 3–4 divisions per day.
- The steady state values obtained using these parameters should be in agreement with our experimental observations on both the total numbers and the composition of BM and transitional B cells. Any parameter set which did not conform to these criteria was automatically rejected.
- The time of arrival to the steady state should be biologically reasonable. That is, since a mouse completes its growth within less than 2 months, parameter sets that resulted in longer times of arrival to the steady state in each subpopulation were also rejected.

These conditions significantly constrain the choice of parameter ranges used in our simulations, such that the parameter subspace which gives results obeying all constraints is rather narrow.

### Model Fitting to the Experimental Data

Our goal here was to check whether the new hypothesis of T3 behavior accounts for B cell dynamics in the spleen), and estimate the parameter ranges characterizing B cell dynamics in the spleen, by fitting simulations to the published data described above. Among all simulations that obeyed the above criteria, we looked for the best fit to the experimental data, defined as the minimum value of the sum of squared deviations of simulated points from experimental data points (a least-squares fit), described by:
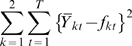
(9)


*Y_kt_* refers to the set of experimental measurements, *f_kt_* refers to the set of simulation results, and these were compared for the two populations (*T_3_* and mature B cells), indexed by *k*, at each time point *t* for which there was an experimental result.

Thus, we searched for parameter values that minimize the deviation of results from experimental data, based on the least-squares criterion defined above. Each automated search involved varying all the relevant parameters simultaneously in very small steps (0.01, or smaller if higher resolution was found to be necessary), recording the fit of each run, and the parameter ranges which gave results within the experimental errors. In order to find whether the model fits the data, we conducted similar searches over all biologically reasonable parameter ranges [24]–[27] for each subpopulation, *T_1/_*_2_, *T*_3_ and the mature B cells, and calculated the fit of those three subpopulations together.

We used “Akaike's Information Criterion” (AIC) to find if our model is more likely to give a good explanation of B cell development in the spleen. We used an adaptation of the AIC method. In this method, we associate an AIC score to the parameter set that minimize the deviation of results from experimental data. We denote M to be the number of parameters fit by the regression, and N to be the number of data points. The AIC score (corrected for small numbers of data points) is thus defined by equation 10.
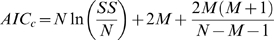
(10)


Suppose AIC_c(A)_ is the score of one with the minimal the deviation of results from experimental data (SS_A_), and AIC_c(B)_ is the score of another set of parameters with a minimal sum SS_B_. In this case, the difference between the AIC_c_ scores is given by equation 11, and is has a negative value, since 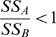.
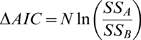
(11)


The probability that we have chosen the correct model (out of those that were considered) is then computed from equation 12. Since we have used the sum of squared deviations as an approximation for the MLE assumed by the AIC criterion, this probability is an approximation.
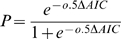
(12)


## Results

### The T3 Pool Represents Both Newly Formed and Mature B Cells Undergoing Anergic Death

Our previously described mathematical models of B cell development in the BM [25], [27] and spleen [26] were used here, but the T1 and T2 B cells were combined into a single subset, because the differences between these two subsets are not important for this study. We later verified that repeating the model fitting to data without combining these 2 subsets gives the same results (not shown).

Our model starts with three bone marrow populations: pro-B [B220^+^CD43^+^IgM^−^], pre-B [B220^+^CD43^−^IgM^−^] and immature B cells [B220+HSA+IgM^hi^IgD^lo^], with cell numbers in these subsets represented by the variables B_o_, B_e_ and B_i_, respectively. However, previous experimental observations distinguish between small, non-cycling cells and large, cycling cells in both the pro-B and pre-B compartments, where the transition from pro-B to pre-B occurs while the cells are cycling. Hence, we break the pro-B and pre-B subsets into two subsets each: B_or_ for small resting pro-B cells (Hardy's fractions A through C) and B_oc_ for large cycling pro-B cells (part of Hardy's fraction C); similarly, B_ec_ for large cycling pre-B cells (the remainder of fraction C) and B_er_ for small resting pre-B cells (fraction D). Immature B (*B*_i_) cells migrate from the bone marrow to the periphery as transitional B cell. The four subpopulations in the periphery are the three transitional B cell subsets and the mature B cell subset in the spleen as defined by Allman et al, [28], Table 1.

In our previous study, we showed that out of 630 possible alternative models, only 8 can explain the population dynamics of transitional B cell differentiation in the spleen [26]. All those modes included the assumption that cells within the T3 subset differentiate into mature B cells; the opposite possibility was not tested in that study. In the present study, we used our previous models of BM (equations 1–8) and spleen populations, but changed the transitional B cell model to include the hypotheses that *T3* subset is characteristic of cells undergoing anergic death, and that at least some of the T3 pool is derived from mature B cells. In order to lower the number of degrees of freedom of the parameter space, we combined *T_1_* and *T_2_* B cells into a single subset, T_1/2_, which later differentiates to *T_3_* and to mature B cells. To examine the hypothesis that the *T3* B cell compartment is the phenotypic niche for cells undergoing negative selection, we ran the equations of the model, in which the differentiation from the mature to the *T3* subset (with rate *δ_m3_*) is added (The inset in Figure 1).

We conducted simulations of this model, using the best set of parameter values previously obtained for the BM equations, and varying the parameters of the spleen populations, in order to obtain the best fit to published experimental data on these subpopulations in mice (section on data for model fitting and ref. [28]).

**Figure 2:**
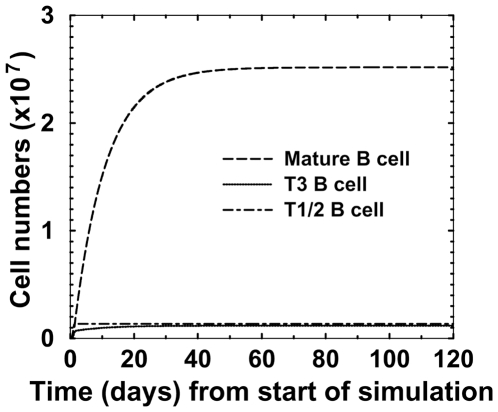
Cell numbers versus time in a simulation of the spleen population model. These numbers were obtained by a simulation with the parameters set that gave the best fit to the data. Parameter values are given in Table 2. The steady-state numbers are: T1/2: 1.17×10^6^ cells, T3: 1.43×10^6^ cells, and Mature FO: 2.51×10^6^ cells.

**Figure 3:**
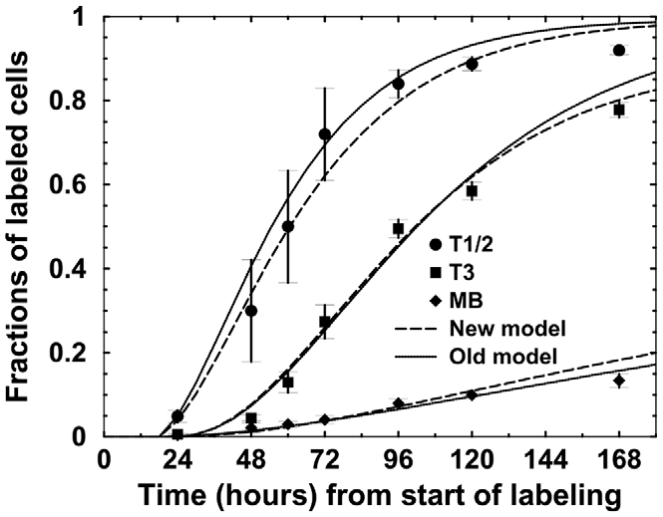
BrdU labeling kinetics. These kinetics were obtained by a simulation of the spleen population model [25]–[26] with the parameter set that gave the best fit to the data [28]. Parameter values are given in Table 2. Simulation results (dashed lines) are presented along with the experimental results (symbols with error bars).

The parameter value ranges for the new hypothesis are presented in Table 2. These are the ranges of parameter values that give results within the experimental range (i.e. the total cell numbers in each population and the fractions of labeled cells within each population are all within the experimental ranges) (Figures 2 and 3). Note that the ranges given here are for each parameter separately. Hence, not all values in the range given for one parameter can necessarily be combined with all values in the range given for other parameters to give acceptable results.

**Table 2:** Parameter ranges that result from the simulation.

Parameter symbol and description a	Value range in acceptable models b	Value in the best-fit model
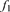 - Fraction of differentiation from BM to T1/2	0.15–0.19	**0.15**
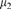 - Death rate of the T1/2 subset	0–0.1	**0.05**
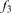 - Fraction of differentiation from BM to T3	0–0.005	**0**
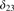 - Differentiation rate from T1/2 to T3	0.07–0.11	**0.1**
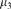 - Death rate of the T3 subset	0.13–0.19	**0.17**
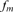 - Fraction of differentiation from BM to mature B	0–0.009	**0.004**
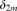 - Differentiation rate from T1/2 to mature B	0.035–0.06	**0.05**
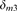 - Differentiation rate from mature B to T3	0.004	0.004
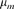 - Death rate of mature B cells	0–0.001	**0**

a Rates are per 6 hours.

b Models that obey our parameter choice criteria and fit the experimental data.

Using Akaike's Information Criterion as described in the methods shows that the probability that we have chosen the correct model is 86%, hence the new hypothesis is more likely to be correct.

We also obtained the same results with separate *T_1_* and *T_2_* B cell subsets as with the combined subsets (data not shown). Thus, we propose that mature naïve B cells undergoing death acquire T3 phenotypic characteristics.

An implication of this proposition is that most of the loss in peripheral B cell maturation may be due to a high rate of loss in the *T_3_* B cell compartment. Indeed, in the parameter value sets that gave the best fit, the death rate of *T_3_* B cells was higher than the death rate in all other splenic subpopulations. The value of the death rate was at least one order of magnitude higher in *T_3_* than of mature B cells, and twice that of the *T_1/2_* subpopulation (Table 2). Therefore we can assume that the throughput of the *T_3_* pool accounts for most of the losses between transitional and mature B cells.

Moreover, in the best-fit simulations, mature B cells differentiate mostly from *T_1_* and *T_2_*, both when we combined them (Table 2) and when we modeled them separately (data not shown). This suggests that *T_3_* is rarely a developmental step in FO B cell maturation. Rather, *T_3_* is only a negatively selected subpopulation.

### The Maximum Fraction of Mature B Cells That Differentiate to the T3 Pool

**Figure 4:**
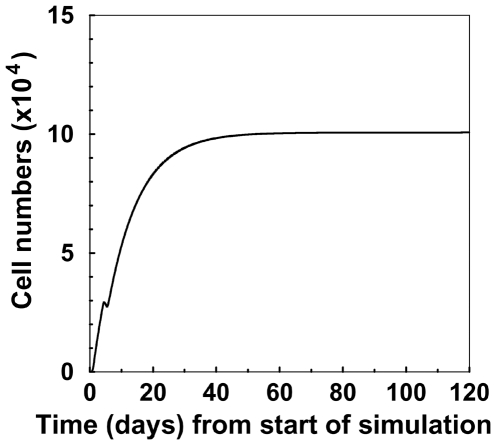
The number of T3 B cells differentiating from the mature compartment per time step. Here we plotted the mature B cell differentiation term (δ_m3_*B_m_) in every time step (6 hours), obtained by a simulation of the new hypothesis. The figure shows the steady state that this number reaches within less than 2 months. Parameter values are given in Table 2.

We next proceeded to estimate the fraction of mature B cells that differentiate to the T3 pool. The range of values of the differentiation rate from mature B cells to *T_3_* B cells (*δ_m3_*) was 0.001–0.004 per 6 hours (Table 2). Multiplying the upper value of this rate by the total number of mature B cells in steady state (2.5×10^7^) in the spleen, we see that the maximum cell number in the *T_3_* pool that could represent recursion from mature B cells at the steady state is around 10^5^ *T_3_* cells per 6 hours (Figure 4). The best-fit value for the mature B cell death rate was zero, so the T3 pool likely contains the vast majority of peripheral B cells–regardless of origin - destined for elimination.

### The Best-Fit Model

In the previous study, several alternative models were found to have a good fit to the data; all of them were models that included a differentiation from T3 to mature B cells and not vice versa, but they differed in the presence or absence of other transitions. For example, we found that the T1 or the T2 stage, or both, may be skipped by a small fraction of the differentiating cells. To find whether all these possibilities are also valid in the new model, we simulated all the alternative models of splenic B cell subsets that include the new hypothesis. Whenever the existence or absence of a certain transition was examined, the range of its rate parameter included the possibility that this rate equals zero. This was applied to *f_1_, f_3_, f_m_, δ_23_, δ_23_*, and *δ_2m_*. Our requirement that a model fit not only the labeling kinetics of all splenic B cell subsets, but also the total cell numbers, was used to reduce the number of acceptable models.

As shown in Table 2, the value ranges for several of these parameters that obey our above-described criteria and fit the experimental data (section 2.4) included zero as a possible value. Our new model fit of the experimental data better than all the alternative models we found in our previous results [26]. The parameter values for the best-fit model are shown in Table 2. Again, the ranges given here are for each parameter separately, and hence not all values in the range given for one parameter can necessarily be combined with all values in the range given for other parameters to give acceptable results. The equations of the best-fit model are the following.
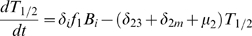
(13)

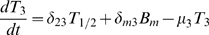
(14)

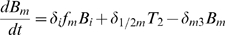
(15)


**Figure 5:**
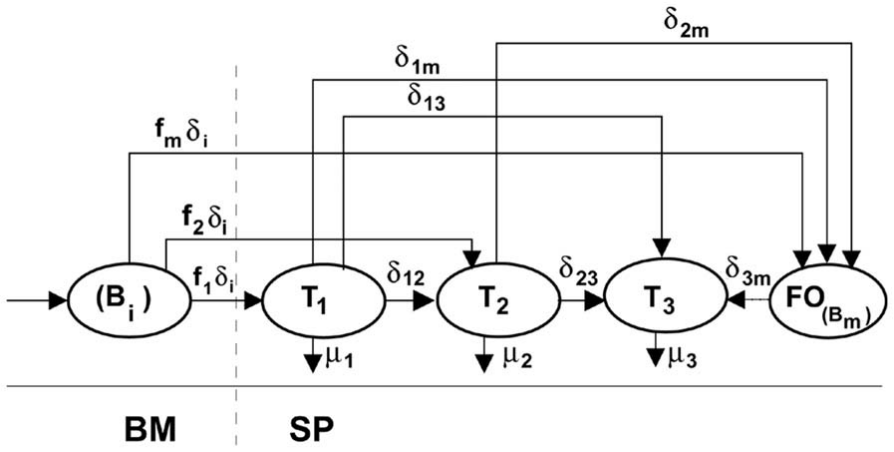
The model without mature B cell death. In this model, mature B cells do not have a death rate, that is μ_m_ = 0. The differentiation from B_m_ to T_3_ occurs a rate of δ_m3_ = 0.003, thus the T_3_ compartment accounts for all the mature B cell turnover. The other parameter values are given in Table 2.

Thus, as shown in Figure 5, the best fit model contains the possibility of some immature B cells “skipping” the peripheral T_1/2_ stages and differentiating directly to mature B cells; T_1/2_ cells may differentiate into either T3 or mature B cells; and, as mentioned above, cell death in the mature B cell compartment is negligible. The death rate in the T3 subset is highest, and this stage is an end stage into which both T_1/2_ and mature B cells may be directed.

## Discussion

This study models the kinetics of splenic B cell compartments, using a combination of in vivo BrdU labeling data [28] and a mathematical model of B cell population dynamics [24]–[27]. Our results suggest the T3 compartment represents a death niche for peripheral B cells, and includes cells from both newly formed and mature compartments undergoing apoptotic death. Thus, under normal steady state conditions, up to 40% of the T3 pool could represent entry from mature pools, with the remainder being derived from newly formed cells. Finally, our analyses are most consistent with a model in which most cells that enter the FO pool do so from the T1 and T2 subsets directly.

We find that the in vivo labeling data are consistent with a model in which the death rate among T_3_ B cells is higher than in any other subpopulations, suggesting this pool represents the principal death niche for peripheral B cells. The model predicts that a majority of T3 B cells are derived from recent marrow émigrés, confirming prior assumptions that the T3 subset contains newly formed B cells that fail to meet the selective criteria imposed during transitional differentiation [31]–[32]. Death during transitional differentiation reflects either the failure to meet a minimum tonic BCR signaling requisite or the onset of anergy from sustained BCR cross-linking. Accordingly, our findings support a model whereby the T3 pool follows a branch-point at which TR cells destined for death versus final maturation have bifurcated [31]. Indeed, the model suggests that the bulk of mature cells arise from the T1/2 pools, with few, if at all, being rescued from the T3 subset [31]–[32].

Our analyses also reveal that up to 40% of the T3 pool, or about 10^5^ T3 B cells, may be derived from the mature B cell compartment. Because the mature pool is numerically large compared to the T3 pool, this indicates a low overall frequency with which mature B cells meet this fate. Nonetheless, it suggests that nearly all mature B cell losses could proceed via this phenotypic intermediate, because the mature B cell turnover of ∼2% per day would generate a steady state value in the 10^5^ range. Thus, B cell losses in the T_3_ compartment can not only account for all losses at the TR to mature B cell checkpoint, but can accommodate the bulk of mature B cell turnover as well. This is consistent with the view that T3 cells represent peripheral B cells destined for death regardless of origin, in accord with recent suggestions from Merrell et al [23].

Together, these findings suggest that B cells fated for imminent elimination from pre-immune subsets comprise the T3 compartment, where they reside briefly. It is tempting to speculate that this reflects a common death pathway, especially since all of these cells rely on continuous signaling via the BCR and BLyS receptor 3 (BR3, also termed BAFFR) to survive. Accordingly, failure to fall within appropriate ranges for signaling via these two systems–regardless of the basis - may lead to acquisition of the T3 phenotype and subsequent death.
